# Idelalisib addition has neutral to beneficial effects on quality of life in bendamustine/rituximab-treated patients: results of a phase 3, randomized, controlled trial

**DOI:** 10.1186/s12955-019-1232-8

**Published:** 2019-11-15

**Authors:** Marco Montillo, Árpád Illés, Tadeusz Robak, Alexander S. Pristupa, Malgorzata Wach, Miklós Egyed, Julio Delgado, Wojciech Jurczak, Franck Morschhauser, Anna Schuh, Herbert Eradat, Sanatan Shreay, Jacqueline C. Barrientos, Andrew D. Zelenetz

**Affiliations:** 1 Department of Hematology, Niguarda Cancer Center, Niguarda Hospital, Piazza Ospedale Maggiore, 3, 20162 Milan, Italy; 2 Department of Hematology, Faculty of Medicine, University of Debrecen, Debrecen, Hungary; 3 Department of Hematology, Medical University of Lodz and Copernicus Memorial Hospital, Lodz, Poland; 4 Department of Hematology, Ryazan Regional Clinical Hospital, Ryazan, Russia; 5 Department Hemato-Oncology and Bone Marrow Transplantation, Medical University of Lublin, Lublin, Poland; 6 Department of Hematology, Somogy County Kaposi Mor Hospital, Kaposvar, Hungary; 7 Department of Hematology, Hospital Clínic, Barcelona, Spain; 8 Maria Sklodowska Curie Institute of Oncology, Krakow, Poland; 9 CHRU Lille, Unité GRITA, Department of Hematology, Université de Lille, Lille, France; 10 University of Oxford, Oxford Cancer and Haematology Centre, Churchill Hospital, Oxford, UK; 11 Division of Hematology-Oncology, David Geffen School of Medicine at UCLA, Los Angeles, CA USA; 12 Gilead Sciences, Inc., Foster City, CA USA; 13 Zucker School of Medicine at Hofstra/Northwell, Hempstead, NY USA; 14 Memorial Sloan Kettering Cancer Center, New York, NY USA

**Keywords:** Idelalisib, Relapsed/refractory CLL, Patient-related outcomes, Health-related quality of life, Randomized phase 3 study

## Abstract

**Background:**

In a phase 3 randomized, double-blind, placebo-controlled trial, treatment with idelalisib, a phosphoinositol-3 kinase δ inhibitor, + bendamustine/rituximab improved progression-free survival (PFS) and overall survival (OS) in adult patients with relapsed/refractory chronic lymphocytic leukemia (R/R CLL). Here we report the results of health-related quality of life (HRQL) analyses from this study.

**Methods:**

From June 15, 2012 to August 21, 2014, 416 patients with R/R CLL were enrolled; 207 patients were randomized to the idelalisib arm and 209 to the placebo arm. In the 416 patients randomized to receive bendamustine/rituximab and either oral idelalisib 150 mg twice-daily or placebo, HRQL was assessed at baseline and throughout the blinded part of the study using the Functional Assessment of Cancer Therapy–Leukemia (FACT-Leu) and EuroQoL Five-Dimension (EQ-5D) visual analogue scale (VAS) questionnaires. The assessments were performed at scheduled patient visits; every 4 weeks for the first 6 months from the initiation of treatment, then every 8 weeks for the next 6 months, and every 12 weeks thereafter until end of study. Least-squares mean changes from baseline were estimated using a mixed-effects model by including treatment, time, and treatment-by-time interaction, and stratification factors as fixed effects. Time to first symptom improvement was assessed by Kaplan-Meier analysis.

**Results:**

In mixed-effects model analysis, idelalisib + bendamustine/rituximab treatment led to clinically meaningful improvements from baseline in leukemia-associated symptoms. Moreover, per Kaplan-Meier analysis, the proportion of patients with symptom improvement was higher and time to improvement was shorter among patients in the idelalisib-containing arm compared with those who did not receive idelalisib. The physical and social/family FACT-Leu subscale scores, along with the self-rated health assessed by EQ-VAS, showed improvement with idelalisib over placebo, but the difference did not reach statistical significance. The functional and emotional FACT-Leu subscale scores remained similar to placebo.

**Conclusions:**

Addition of idelalisib to bendamustine/rituximab, apart from improving PFS and OS, had a neutral to beneficial impact on HRQL in patients with R/R CLL, particularly by reducing leukemia-specific disease symptoms.

**Trial registration:**

Clinicaltrials.gov NCT01569295. Registered April 3, 2012.

## Background

In 2018, chronic lymphocytic leukemia (CLL)—the most common chronic leukemia—represented an estimated 1.2% of all newly diagnosed cancers in the US, with an estimated 20,940 new CLL cases and 4510 deaths [1]. The age-adjusted rate of CLL incidence is 4.7 per 100,000 persons per year. Owing to the introduction of new, improved treatment regimens, the 5-year relative survival rate increased from 65.5% in 1975 to 84.2% in 2014 [1]. Despite this improvement, CLL remains incurable, and the majority of patients experience disease relapse [2]. The risk of relapse is increased in patients with CLL-associated genomic aberrations [3–6], patients ≥65 years with comorbid conditions [7–10], and refractory disease [11]. It is not uncommon for these patients to experience low treatment satisfaction and greatly reduced health-related quality of life (HRQL), mostly due to disease-related symptoms, toxicity of therapies, and anxiety associated with relapsing disease [12–14]. Despite recommendations to include prospective analyses of patient-reported outcomes (PROs) as additional endpoints in oncology clinical trials [15–17], PRO-based reports on HRQL in CLL are sparse. Patient-reported outcomes data can provide important information on the impact of new treatment regimens, including their efficacy and toxicity, as seen from the patient’s perspective [16], and improvement in HRQL often reflects the efficacy of the new treatment under evaluation [18–21].

Idelalisib, in combination with rituximab, was approved for the treatment of patients with relapsed CLL for whom rituximab alone would be considered an appropriate therapy due to other comorbidities [22]. To examine the usefulness of idelalisib in additional clinical scenarios, we conducted a pivotal phase 3, randomized, multicenter, double-blind, placebo-controlled trial of idelalisib, a phosphoinositol-3 kinase δ (PI3Kδ) inhibitor, in combination with bendamustine and rituximab, in patients with relapsed/refractory CLL (R/R CLL). The efficacy of the idelalisib/bendamustine/rituximab combination in this trial was superior to placebo/bendamustine/rituximab, and substantially improved the primary endpoint of progression-free survival (PFS) as well as the key secondary endpoint of overall survival (OS) [23].

Prespecified exploratory endpoints in this study included assessment of HRQL using the Functional Assessment of Cancer Therapy – Leukemia (FACT-Leu) validated questionnaire [24–28]. The FACT-Leu was developed as a disease-specific HRQL questionnaire for patients with leukemia [27, 28], and is composed of subscales scoring the patient’s physical, functional, social/family, and emotional well-being, as well as leukemia-specific disease symptoms [27, 28]. Another prespecified exploratory endpoint was global health status and self-rated health assessed using the EuroQoL Five-Dimension (EQ-5D) visual analogue scale (VAS) questionnaires [29, 30]. We present the results of these prospectively defined analyses comparing the impact of treatment with idelalisib combined with bendamustine/rituximab with that of bendamustine/rituximab/placebo, on patients’ HRQL.

## Methods

### Study design and participants

This was a phase 3, randomized, multicenter, double-blind, placebo-controlled study (NCT01569295) in adult patients who were diagnosed with CLL requiring treatment according to International Workshop on Chronic Lymphocytic Leukemia criteria [17]. Eligible patients had measurable lymphadenopathy by computed tomography or magnetic resonance imaging, received prior therapy containing a purine analog or bendamustine and an anti-CD20 monoclonal antibody, experienced CLL progression within <36 months since completion of the last prior therapy, were eligible to receive cytotoxic therapy, and had a Karnofsky Performance Status score of ≥60. Study design and detailed eligibility criteria were published previously [23].

### Ethics, consent, and permissions

The study protocols were approved by the Institutional Review Boards at each study site. The trial was conducted according to the principles of Good Clinical Practice and the Declaration of Helsinki. All patients provided written informed consent.

### Treatments and endpoints

Patients received oral idelalisib 150 mg or matching placebo twice daily. Bendamustine 70 mg/m^2^ was administered intravenously on days 1 and 2 for six 28-day cycles in both arms. Rituximab was administered intravenously with each cycle of bendamustine at 375 mg/m^2^ on day 1 of cycle 1, and 500 mg/m^2^ on day 1 of cycles 2 to 6. Idelalisib/placebo was given continuously, until disease progression, death, or intolerable toxicity. Bendamustine and rituximab were administered for up to a maximum of 12 and 6 infusions, respectively [23].

The details on primary and secondary endpoints have been reported [23]. Briefly, the primary endpoint was PFS; OS, overall response rate, and safety were among the key secondary endpoints. The prespecified HRQL-related exploratory endpoints were change from baseline in HRQL domain and symptom scores based on the FACT-Leu, and change from baseline in overall health and single-item dimension scores from the EQ-5D and EQ-VAS questionnaires. Key time points were the end of the randomized, double-blind initial period of combination therapy with bendamustine/rituximab at week 24 and the end of the preplanned continuing therapy period with idelalisib or placebo alone at week 48.

### HRQL assessments

Patient well-being was assessed using the FACT-Leu questionnaire composed of 44 items measuring physical well-being (PWB, 7 items), functional well-being (FWB, 7 items), social/family well-being (S/FWB, 7 items), emotional well-being (EWB, 6 items), and leukemia-specific concerns (LeuS, 17 items) [28], scored based on the Functional Assessment of Chronic Illness Therapy-3 scoring guideline and user manual [31]. The subscale scores represent the sums of each individual item score. The composite scores include FACT-Leu total score (range 0–176), which is the sum of all subscales, and the Trial Outcome Index (TOI, range 0–124), which is the sum of the PWB, FWB, and LeuS subscales. Higher scores are associated with better self-reported HRQL.

The EQ-5D questionnaire contains 5 dimensions: mobility, self-care, usual activities, pain/discomfort, and anxiety/depression. Each dimension has 3 levels: no problems, some problems, and extreme problems [32]. The EQ-5D is converted into a single utility index, designed as an international, standardized questionnaire for evaluation of HRQL [29, 30], by applying the US preference-weighted index [33]. The EQ-VAS is used to assess patient’s self-rated health on a 100-mm scale ranging from “Worst imaginable health state” to “Best imaginable health state.” The EQ-5D utility and EQ-VAS scores are considered reliable and valid for assessing HRQL in cancer patients [34]. Positive changes from baseline indicate improvement in HRQL.

The surveys were administered every 4 weeks for the first 6 months from the initiation of treatment, then every 8 weeks for the next 6 months, and every 12 weeks thereafter until the end of study or until the patient was no longer receiving blinded study drug for any reason.

### HRQL statistical analyses

The FACT Leu and EQ-5D questionnaire was scored and processed according to the user manual [32]. The HRQL questionnaire compliance was defined as a patient having answered at least 1 question at an assessment time point. The compliance rates for each study arm and at each time point were calculated as the number of patients who completed at least 1 question divided by the total number of patients available at that assessment time point. The frequency and proportion of reported problems for each of the five EQ-5D dimensions were summarized at each scheduled assessment. For the FACT-Leu, EQ-VAS questionnaires, and EQ-5D utility index, least-squares mean changes from baseline were estimated using a mixed-effects model, by including treatment, time, and treatment-by-time interaction, and stratification factors as fixed effects. Mixed-effects model used all available data up to week 84, as < 10% of placebo patients have data available beyond week 84. The least-squares means of change from baseline over time were plotted. The minimally important difference (MID) ranges were defined for the different subscales and are summarized in Additional file 1: Table S1. The lower bound of the MID range was utilized when defining symptom improvement; an increase of at least 3 points from baseline for PWB, S/FWB, FWB, and EWB, and 5 points for LeuS (reaching MID). Time to first symptom improvement was analyzed by the Kaplan-Meier (KM) method. The hazard ratios with 95% confidence intervals (CIs) were estimated from a Cox proportional hazards model without any adjustment and *P*-value from log-rank test was reported to examine the difference between the 2 treatment arms.

The HRQL analyses were based on the intent-to-treat analysis set, which included all patients randomized in the study regardless of whether study drug was administered, and with treatment groups designated according to initial randomization. Nominal *P*-value threshold of 0.05 was used for significance testing without multiplicity adjustment. All the analyses were performed using SAS version 9.2 (Cary, North Carolina).

## Results

### Key patient characteristics

From June 15, 2012, to August 21, 2014, 416 patients with R/R CLL were enrolled; 207 patients were randomized to the idelalisib arm and 209 to the placebo arm. Based on the results of the formal interim analysis performed in June 2015, the Independent Data Monitoring Committee recommended study unblinding. Although the study is still ongoing, the HRQL data presented herein reflect a data cutoff date of October 7, 2015, at the time of study unblinding. At the time of this analysis, 141 (34%) of 416 patients were continuing in the study; 95 (46%) of 207 in the idelalisib group and 46 (22%) of 209 in the placebo group.

Patient disposition and demographic and baseline characteristics were published previously [23]. The patients received a median (quartile [Q1, Q3]) of 6 (4, 6) cycles of treatment with bendamustine and 6 (5, 6) with rituximab; the median (Q1, Q3) exposure to idelalisib and placebo was 14.8 (5.9, 18.9) months and 11.1 (5.8, 15.3) months, respectively. Generally, demographic and baseline disease characteristics were balanced across the treatment groups [23].

### Summary of the efficacy and safety results

The detailed efficacy and safety results were previously reported at the data cutoff of October 7, 2015 [23], the same as the present analysis. The addition of idelalisib to bendamustine/rituximab led to a substantial improvement in the efficacy of the treatment, compared with bendamustine/rituximab alone [23]. The most common all-grade adverse events (AEs) were neutropenia and pyrexia in the idelalisib arm and neutropenia and nausea in the placebo arm [23].

### Patient-reported FACT-Leu and EQ-5D outcomes

Among patients randomized to idelalisib and placebo arms, similar numbers of patients were available for HRQL analyses at baseline (Table 1). Mean baseline scores for the FACT-Leu and EQ-5D questionnaires were also comparable between the treatment arms (Table 1).

**Table 1:** Scores for the HRQL questionnaires at baseline

	Idelalisib/rituximab/bendamustine, *N* = 207	n	Placebo/rituximab/bendamustine, *N* = 209	n
FACT-Leu total score^a^	125.27 (24.103)	196	123.17 (27.540)	202
Trial outcome index score^b^	86.12 (18.662)	196	84.71 (21.337)	203
Physical well-being	21.77 (5.012)	197	21.39 (5.418)	203
Social/family well-being	21.51 (5.502)	198	21.40 (5.392)	203
Emotional well-being	17.68 (4.215)	198	16.93 (4.902)	204
Functional well-being	17.99 (6.068)	199	17.32 (6.135)	204
Leukemia-specific symptoms	46.31 (10.312)	199	45.95 (12.206)	204
EQ-5D utility index	0.78 (0.217)	197	0.78 (0.228)	195
EQ-VAS	68.8 (17.81)	190	67.4 (19.28)	194

Analyzed in the ITT population. All data presented as mean (SD); n indicates number of patients available for HRQL assessment at baseline

*EQ-5D* EuroQoL Five-Dimension, *FACT-Leu* functional assessment of cancer therapy–leukemia, *EWB* emotional well-being, *FWB* functional well-being, *HRQL* health-related quality of life, *ITT* intent-to-treat, *LeuS* leukemia-specific concerns, *PWB* physical well-being, *S/FWB* social/family well-being, *SD* standard deviation, *TOI* trial outcome index, *VAS* visual analog scale

^a^FACT-Leu Total = LeuS + PWB + S/FWB + EWB + FWB

^b^TOI = LeuS + PWB + FWB

Assessments of compliance for the FACT-Leu and EQ-5D questionnaires were conducted over the initial 144 weeks of study. Compliance rates for both FACT-Leu and EQ-5D were high and exceeded 80% during the first 120 weeks of study in both treatment arms (Additional file 1: Tables S2 and S3).

#### FACT-Leu

**Fig. 1:**
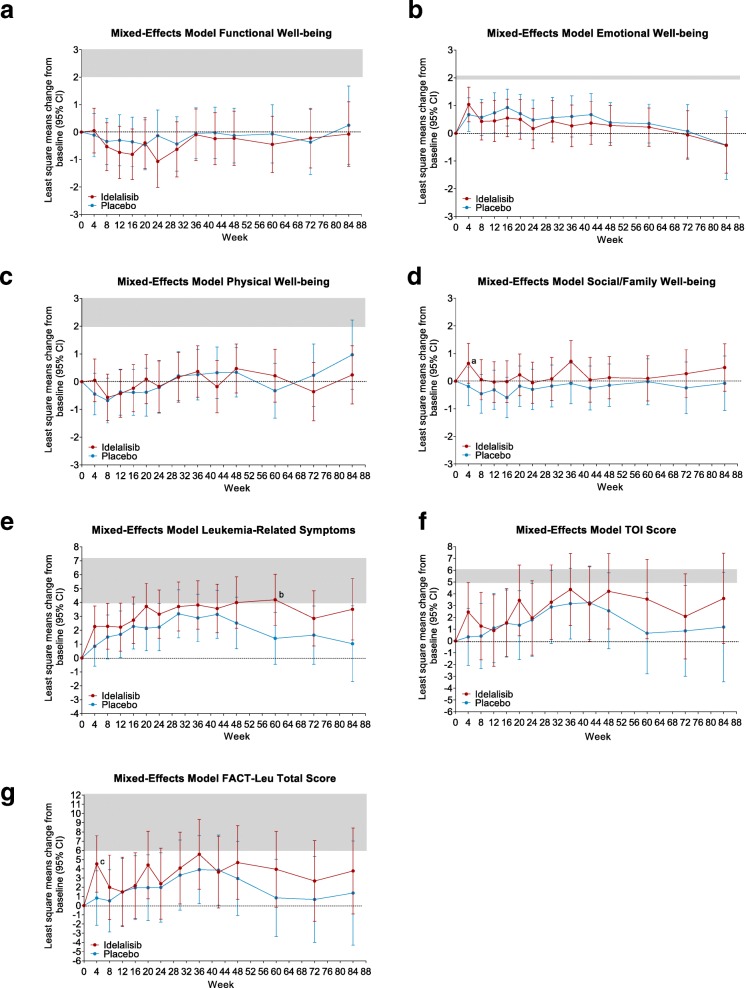
Mixed-effects model analysis of FACT-Leu. **a**, FWB; **b**, EWB; **c**, PWB; **d**, S/FWB; **e**, LeuS; **f**, TOI score; **g**, FACT-Leu total score. Curves above the x-axis indicate positive effects, and curves below the axis show negative effects. Gray area denotes MID range. ^a^*P* = 0.0525 for treatment difference. ^b^*P* = 0.0192 for treatment difference. ^c^*P* = 0.0343 for treatment difference. *CI* confidence interval, *EWB* emotional well-being, *FACT-Leu* Functional Assessment of Cancer Therapy–Leukemia, *FWB* functional well-being, *LeuS* leukemia-specific symptoms, *MID* minimally important difference, *PWB* physical well-being, *S/FWB* social/family well-being, *TOI* trial outcome index

The addition of idelalisib to bendamustine/rituximab had no significant impact on FWB, EWB, and PWB compared with placebo/bendamustine/rituximab (Fig. 1a, b, c). In contrast, idelalisib increased the S/FWB and LeuS subscale scores, as well as composites TOI and FACT Leu total score, compared with the placebo arm, indicating an improvement in HRQL in idelalisib-treated patients (Fig. 1d, e, f, g). In a mixed-effects model including treatment arm (idelalisib vs placebo) and duration, treatment arm had no significant fixed effect on any FACT-Leu score. The effect of treatment duration was significant for FACT-Leu total score (*P* = 0.0076), TOI score *(P* = 0.0103), PWB (*P* = 0.0017), EWB (*P* = 0.0142), and LeuS (*P* = 0.0023); there was no significant interaction between treatment arm and duration for any FACT-Leu score. The treatment difference for idelalisib vs placebo was statistically significant for the LeuS subscale at week 60 (*P* = 0.0192), and for S/FWB and FACT-Leu total score at week 4 (*P* = 0.0525 and *P* = 0.0343, respectively) (Fig. 1, Additional file 1: Table S4). The least squares mean changes from baseline in the LeuS subscale scores were within the MID range of 4 to 7 during the period between week 48 and week 60 in the idelalisib arm, while the values in the placebo arm did not reach the MID at any time points (Fig. 1e).

**Fig. 2:**
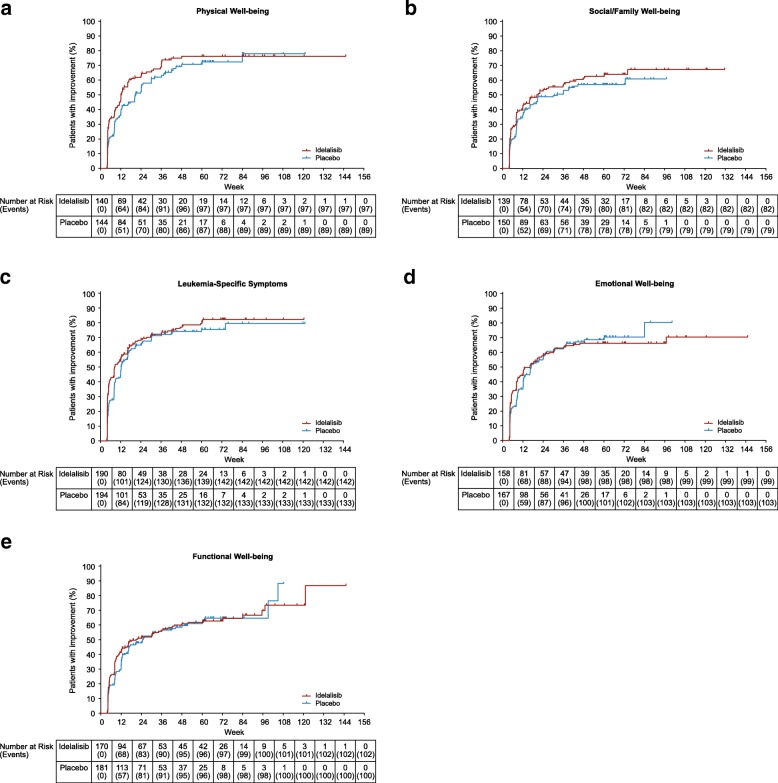
Percent of patients with improvements in FACT-Leu subscales (Kaplan-Meier analysis). **a**, PWB; **b**, S/FWB; **c**, LeuS; **d**, EWB; **e**, FWB. *EWB* emotional well-being, *FACT-Leu* Functional Assessment of Cancer Therapy–Leukemia, *FWB* functional well-being, *LeuS* leukemia-specific symptoms, *PWB* physical well-being, *S/FWB* social/family well-being

The KM analysis of symptom improvement suggests that an increased number of patients with the MID improvement, as well as shorter time to symptom improvement, were reported for the 5 FACT-Leu subscales in the idelalisib-containing arm compared with the placebo arm, but none of the differences reached statistical significance (Table 2). A higher proportion of patients treated with idelalisib achieved improvement in PWB, S/FWB and LeuS subscale scores, but not EWB and FWB scores, compared with patients treated with placebo (Fig. 2a–e). Overall, addition of idelalisib to bendamustine/rituximab had a neutral to numerically favorable effect on change from baseline in FACT-Leu scores of patients with R/R CLL.

**Table 2:** Summary of symptom improvement

	Idelalisib/bendamustine/rituximab, N = 207			Placebo/bendamustine/rituximab, N = 209				
	Patients with MID improvement^a^	Time to symptom improvement^b^	Proportion of patients with any symptom improvement^c^	Patients with MID improvement^a^	Time to symptom improvement^b^	Proportion of patients with any symptom improvement^c^	HR (95% CI)	*P*-value
PWB	97 (69.3)	12.3 (9.1, 16.1) *N* = 140	139 (67.1)	89 (61.8)	20.9 (12.9, 30.1) *N* = 144	141 (67.5)	1.28 (0.96, 1.70)	0.1026
S/FWB	82 (59.0)	20.4 (12.1, 39.9) *N* = 139	130 (62.8)	79 (52.7)	32.4 (16.3, 72.7) *N* = 150	139 (66.5)	1.20 (0.88, 1.63)	0.2663
EWB	99 (62.7)	16.1 (8.9, 23.9) *N* = 158	159 (76.8)	103 (61.7)	16.9 (12.4, 24.4) *N* = 167	147 (70.3)	1.04 (0.79, 1.37)	0.8357
FWB	102 (60.0)	20.9 (12.1, 39.9) *N* = 170	142 (68.6)	100 (55.2)	24.7 (16.1, 44.3) *N* = 181	145 (69.4)	1.07 (0.81, 1.42)	0.6321
LeuS	142 (74.7)	8.4 (6.3, 12.7) *N* = 190	168 (81.2)	133 (68.6)	12.3 (11, 16.3) *N* = 194	168 (80.4)	1.22 (0.96, 1.55)	0.1134

Analyzed in the ITT population. Patients with baseline PWB/S/FWB/FWB > 25, EWB > 21, and LeuS > 63 are not included in the respective analysis of improvement

*CI* confidence interval, *EWB* emotional well-being, *FWB* functional well-being, *HRQL* health-related quality of life, *HR* hazard ratio, *ITT* intent-to-treat, *LeuS* leukemia-specific concerns, *MID* minimally important difference, *PWB* physical well-being, *S/FWB* social/family well-being

^a^Data presented as n (%). MID symptom improvement was defined as an increase of ≥3 points from baseline for PWB/S/FWB/FWB/EWB and 5 points for LeuS

^b^Data presented as median (95% CI), weeks. Patients who did not experience a symptom improvement compared to baseline were censored at their last available HRQL assessment time. Time to symptom improvement (weeks) = (date of first symptom improvement − date of randomization + 1)/7

^c^Data presented as n (%). Patients with any increase from baseline

#### EQ-5D

**Fig. 3:**
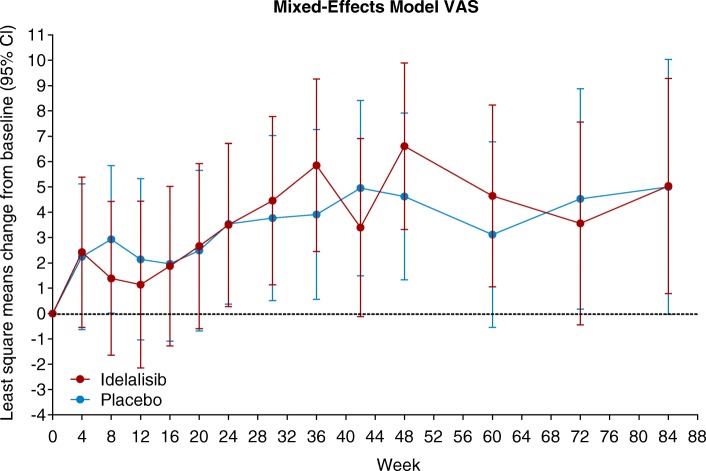
Mixed-effects model analysis of EuroQoL Five-Dimension VAS. *CI* confidence interval, *VAS* visual analogue scale

Proportions of patients reporting level 1 or 2 EQ-5D health scores at weeks 24 and 48 were generally similar between the idelalisib and placebo treatment arms. Slightly fewer idelalisib-treated patients reported extreme problems (level 3) with anxiety/depression and pain/discomfort at baseline and after 24 and 48 weeks of treatment compared with patients who received placebo (Table 3). The EQ-5D utility index and EQ-VAS showed trends toward improvement with idelalisib relative to placebo treatment, but these did not reach significance (Table 4, Fig. 3). In a mixed-effects model including treatment (idelalisib vs placebo) and treatment duration, treatment duration had a significant fixed effect for EQ-5D utility index (*P* = 0.0169) and EQ-VAS (*P* = 0.0061); treatment had no significant fixed effect on either score, but there was a significant fixed effect of the interaction between treatment and treatment duration on EQ-5D utility index *(P* = 0.0395). Combination therapy with idelalisib vs placebo did not worsen patient-reported health—and resulted in favorable trends in some measures—in patients with R/R CLL treated with bendamustine/rituximab.

**Table 3:** Summary of EQ-5D questionnaire by dimension

	Idelalisib/rituximab/bendamustine, *N* = 207			Placebo/rituximab/bendamustine, *N* = 209		
Dimensions	Baseline	Week 24	Week 48	Baseline	Week 24	Week 48
Anxiety/Depression						
Level 1	113 (57.1)	102 (65.8)	85 (66.4)	117 (59.1)	91 (60.3)	70 (66.7)
Level 2	84 (42.4)	50 (32.3)	41 (32.0)	75 (37.9)	55 (36.4)	32 (30.5)
Level 3	1 (0.5)	3 (1.9)	2 (1.6)	6 (3.0)	5 (3.3)	3 (2.9)
Mobility						
Level 1	145 (73.6)	119 (76.8)	94 (73.4)	142 (71.4)	112 (74.7)	84 (80.0)
Level 2	52 (26.4)	36 (23.2)	34 (26.6)	55 (27.6)	38 (25.3)	21 (20.0)
Level 3	0	0	0	2 (1.0)	0	0
Pain/Discomfort						
Level 1	105 (53.3)	96 (61.9)	77 (60.2)	114 (57.0)	79 (52.3)	63 (60.6)
Level 2	85 (43.1)	54 (34.8)	51 (39.8)	81 (40.5)	69 (45.7)	37 (35.6)
Level 3	7 (3.6)	5 (3.2)	0	5 (2.5)	3 (2.0)	4 (3.8)
Self-Care						
Level 1	184 (92.9)	136 (87.7)	115 (89.8)	184 (92.5)	131 (86.8)	92 (87.6)
Level 2	14 (7.1)	19 (12.3)	13 (10.2)	13 (6.5)	20 (13.2)	12 (11.4)
Level 3	0	0	0	2 (1.0)	0	1 (1.0)
Usual Activities						
Level 1	126 (63.6)	93 (60.4)	83 (64.8)	122 (61.3)	87 (57.6)	71 (68.3)
Level 2	65 (32.8)	52 (33.8)	45 (35.2)	70 (35.2)	61 (40.4)	31 (29.8)
Level 3	7 (3.5)	9 (5.8)	0	7 (3.5)	3 (2.0)	2 (1.9)

Analyzed in the ITT population. All data represented as n (%)

Level 1: no problems; Level 2: some problems; Level 3: extreme problems

*EQ-5D* EuroQoL Five-Dimension, *ITT* intent-to-treat

**Table 4:** Mixed-effects model analysis for functional assessment of cancer therapy using EQ-5D in the ITT population

Treatment difference^a^ LSM (95% CI)		
	EQ-5D UI	EQ-VAS
Week 4	0.03 (−0.01, 0.07)	0.18 (−3.24, 3.61)
*P*-value	0.1302	0.9167
Week 8	−0.01 (−0.05, 0.04)	−1.54 (−5.06, 1.98)
*P*-value	0.7570	0.3895
Week 12	0.00 (−0.04, 0.05)	−1.00 (−4.96, 2.97)
*P*-value	0.8730	0.6216
Week 16	0.02 (−0.03, 0.07)	−0.09 (−3.82, 3.65)
*P*-value	0.4325	0.9631
Week 20	0.02 (−0.03, 0.06)	0.17 (−3.74, 4.09)
*P*-value	0.4514	0.9301
Week 24	0.04 (−0.01, 0.08)	−0.04 (−3.92, 3.85)
*P*-value	0.1433	0.9851
Week 30	0.03 (−0.02, 0.08)	0.69 (−3.36, 4.73)
*P*-value	0.2915	0.7389
Week 36	0.02 (−0.03, 0.07)	1.94 (−2.24, 6.13)
*P*-value	0.4630	0.3623
Week 42	0.00 (−0.06, 0.06)	−1.56 (−5.92, 2.80)
*P*-value	0.9995	0.4823
Week 48	0.02 (−0.04, 0.07)	1.99 (−2.06, 6.03)
*P*-value	0.5601	0.3348
Week 60	0.03 (−0.03, 0.09)	1.53 (−3.05, 6.11)
*P*-value	0.2981	0.5128
Week 72	0.04 (−0.04, 0.11)	−0.97 (−6.41, 4.48)
*P*-value	0.3131	0.7275
Week 84	−0.04 (− 0.11, 0.02)	0.03 (−6.14, 6.20)
*P*-value	0.2039	0.9921

*CI* confidence interval, *EQ-5D* EuroQoL Five-Dimension, *UI* utility index, *ITT* intent-to-treat, *LSM* least squares means, *VAS* visual analog scale

## Discussion

In the primary analysis, the addition of idelalisib to bendamustine/rituximab resulted in superior PFS and OS compared with bendamustine and rituximab alone, thus providing a substantial clinical benefit to R/R CLL patients—a patient population that is difficult to treat [23]. Apart from treatment goals, a more holistic understanding of the impact of treatment including patients’ quality of life is also important, and HRQL data constitutes an important part of treatment evaluation. Because patients in both the idelalisib and placebo arms received concurrent chemo-immunotherapy with associated side effects, idelalisib could not be expected to substantially improve quality of life (QoL) compared with placebo. However, there was potential concern that addition of idelalisib to bendamustine/rituximab would adversely affect change in QoL relative to treatment with bendamustine/rituximab alone.

These prospectively defined HRQL analyses indicate that idelalisib in combination with bendamustine/rituximab treatment had a neutral impact on several HRQL domains in patients with R/R CLL compared with bendamustine/rituximab alone. Per mixed-effects model analyses, the addition of idelalisib to bendamustine/rituximab did not affect the FWB and EWB subscale scores, but provided minor improvements in the PWB, S/FWB, and LeuS subscale scores as well as the FACT-Leu composite measures TOI and FACT-Leu total score. Increased LeuS subscale scores in the idelalisib arm were within the MID range at weeks 48 and 60 and reached statistical significance at week 60. The KM analysis of the symptom improvement showed that among idelalisib-treated patients, time to symptom improvement was shortened by approximately 4 to 12 weeks for the LeuS, PWB, and S/FWB subscales, compared with the placebo arm, and a higher proportion of patients had clinically meaningful improvements in these HRQL domains.

Idelalisib treatment led to small numerical improvements in HRQL in the global health status, as evidenced by the EQ-5D results, particularly reduction of the number of patients who perceived themselves to experience “extreme problems” within the anxiety/depression and pain/discomfort dimensions. The mean changes from baseline in self-rated health, as assessed by EQ-VAS, was also improved, and reached statistical significance vs placebo at week 36 when patients had stopped concurrent chemo-immunotherapy. The rather small differences in overall quality of life observed between the 2 treatment arms may be due to side effects from the concurrent chemo-immunotherapy exerting a dominant effect on quality of life measurements. However, even the slight QoL improvement in patients treated with idelalisib vs placebo in addition to bendamustine/rituximab is valuable information considering the AEs recorded in previous clinical trials of idelalisib [23, 35, 36].

Recently published analyses of PROs assessed in phase 3 randomized trials in patients with relapsed CLL reported that ibrutinib in combination with bendamustine/rituximab vs bendamustine/rituximab alone neither improved nor adversely impacted HRQL [37], and small positive improvements were noted with the addition of ofatumumab to chlorambucil [38] or to fludarabine and cyclophosphamide [21]. However, apart from the fact that comparisons of the results across different studies have several limitations, even indirect comparison of these findings with the results from our study is difficult because of different tools used for PRO evaluations (The Functional Assessment of Chronic Illness Therapy - Fatigue scale and European Organisation for Research and Treatment of Cancer Quality of Life Questionnaire, in the ibrutinib and ofatumumab studies, and FACT-Leu in this study).

## Conclusion

In summary, in patients with incurable diseases such as CLL, treatment efficacy needs to be balanced with safety and HRQL. In this study, we found that adding idelalisib to bendamustine/rituximab—even for those patients already treated with a prior bendamustine-containing regimen—improved treatment efficacy with, importantly, a neutral or beneficial impact on patients’ HRQL. These HRQL findings provide additional information to balance the improved efficacy of the combination regimen with possible safety concerns reported with idelalisib use [23]. Overall, these results further support the existing body of evidence indicating that idelalisib treatment benefits patients with R/R CLL.

## Supplementary information

**Additional file 1: Table S1.** Questionnaires used to assess health-related quality of life. **Table S2.** Compliance rates: FACT-Leu questionnaire. **Table S3.** Compliance rates: EQ-5D questionnaire. **Table S4.** Mixed-effects model analysis estimates (idelalisib/placebo) for functional assessment of cancer therapy using FACT-Leu.

## Data Availability

The data that support the findings of this study are available from Gilead Sciences, Inc., but restrictions apply to the availability of these data, which were used under license for the current study, and so are not publicly available. Data are however available from the authors upon reasonable request and with permission of Gilead Sciences, Inc.

## Abbreviations

AE: Adverse event
CI: Confidence interval
CLL: Chronic lymphocytic leukemia
EQ-5D: EuroQoL Five-Dimension
EWB: Emotional well-being
FACT-Leu: Functional assessment of cancer therapy – leukemia
FWB: Functional well-being
HR: Hazard ratio
HRQL: Health-related quality of life
KM: Kaplan-Meier
LeuS: Leukemia-specific concerns
MID: Minimally important difference
OS: Overall survival
PFS: Progression-free survival
PI3Kδ: Phosphoinositol-3 kinase δ
PRO: Patient-reported outcomes
PWB: Physical well-being
Q: Quartile
QoL: Quality of life
R/R: Relapsed/refractory
S/FWB: Social/family well-being
TOI: Trial outcome index
VAS: Visual analog scale

## References

[1] SEER Cancer statistics review, 1975–2015. Available at https://seer.cancer.gov/statfacts/html/clyl.html. Accessed 22 Oct 2018.

[2] Chronic Lymphocytic Leukemia/Small Lymphocytic Lymphoma. NCCN Clinical Practice Guidelines in Oncology 2017;Version I.2017-September 28, 2016.

[3] Badoux XC, Keating MJ, Wierda WG (2011). What is the best frontline therapy for patients with CLL and 17p deletion?. Curr Hematol Malig Rep.

[4] Dicker F, Herholz H, Schnittger S, Nakao A, Patten N, Wu L (2009). The detection of TP53 mutations in chronic lymphocytic leukemia independently predicts rapid disease progression and is highly correlated with a complex aberrant karyotype. Leukemia..

[5] Dohner H, Stilgenbauer S, Benner A, Leupolt E, Krober A, Bullinger L (2000). Genomic aberrations and survival in chronic lymphocytic leukemia. N Engl J Med.

[6] Stilgenbauer S, Sander S, Bullinger L, Benner A, Leupolt E, Winkler D (2007). Clonal evolution in chronic lymphocytic leukemia: acquisition of high-risk genomic aberrations associated with unmutated VH, resistance to therapy, and short survival. Haematologica..

[7] Shanafelt T (2013). Treatment of older patients with chronic lymphocytic leukemia: key questions and current answers. Hematol Am Soc Hematol Educ Program.

[8] Smolej L (2010). How I treat elderly or comorbid patients with chronic lymphocytic leukemia. Acta Med (Hradec Kralove).

[9] Smolej L (2012). Therapy of elderly/comorbid patients with chronic lymphocytic leukemia. Curr Pharm Des.

[10] National Cancer Institute: Surveillance Epidemiology and End Results (SEER) program. SEER Stat Fact Sheets: Chronic Lymphocytic Leukemia; 2011. Available at http://seer.cancer.gov/statfacts/html/clyl.html. Accessed 12 Feb 2016.

[11] Veliz M, Pinilla-Ibarz J (2012). Treatment of relapsed or refractory chronic lymphocytic leukemia. Cancer Control.

[12] Shanafelt TD, Bowen D, Venkat C, Slager SL, Zent CS, Kay NE (2007). Quality of life in chronic lymphocytic leukemia: an international survey of 1482 patients. Br J Haematol.

[13] Holtzer-Goor KM, Schaafsma MR, Joosten P, Posthuma EF, Wittebol S, Huijgens PC (2015). Quality of life of patients with chronic lymphocytic leukaemia in the Netherlands: results of a longitudinal multicentre study. Qual Life Res.

[14] Molica S (2005). Quality of life in chronic lymphocytic leukemia: a neglected issue. Leuk Lymphoma..

[15] Basch E, Abernethy AP, Mullins CD, Reeve BB, Smith ML, Coons SJ (2012). Recommendations for incorporating patient-reported outcomes into clinical comparative effectiveness research in adult oncology. J Clin Oncol.

[16] Garcia SF, Cella D, Clauser SB, Flynn KE, Lad T, Lai JS (2007). Standardizing patient-reported outcomes assessment in cancer clinical trials: a patient-reported outcomes measurement information system initiative. J Clin Oncol.

[17] Hallek Michael, Cheson Bruce D., Catovsky Daniel, Caligaris-Cappio Federico, Dighiero Guillaume, Döhner Hartmut, Hillmen Peter, Keating Michael J., Montserrat Emili, Rai Kanti R., Kipps Thomas J. (2008). Guidelines for the diagnosis and treatment of chronic lymphocytic leukemia: a report from the International Workshop on Chronic Lymphocytic Leukemia updating the National Cancer Institute–Working Group 1996 guidelines. Blood.

[18] Cannella L, Caocci G, Jacobs M, Vignetti M, Mandelli F, Efficace F (2015). Health-related quality of life and symptom assessment in randomized controlled trials of patients with leukemia and myelodysplastic syndromes: what have we learned?. Crit Rev Oncol Hematol.

[19] Catovsky D, Richards S, Matutes E, Oscier D, Dyer MJ, Bezares RF (2007). Assessment of fludarabine plus cyclophosphamide for patients with chronic lymphocytic leukaemia (the LRF CLL4 trial): a randomised controlled trial. Lancet..

[20] Eichhorst BF, Busch R, Obwandner T, Kuhn-Hallek I, Herschbach P, Hallek M, German CLL Study Group. Health-related quality of life in younger patients with chronic lymphocytic leukemia treated with fludarabine plus cyclophosphamide or fludarabine alone for first-line therapy: a study by the German CLL Study Group. J Clin Oncol. 2007;25:1722–31. 10.1200/JCO.2006.05.6929 17389338

[21] Robak T, Warzocha K, Govind Babu K, Kulyaba Y, Kuliczkowski K, Abdulkadyrov K (2017). Health-related quality of life and patient-reported outcomes of ofatumumab plus fludarabine and cyclophosphamide versus fludarabine and cyclophosphamide in the COMPLEMENT 2 trial of patients with relapsed CLL. Leuk Lymphoma..

[22] ZYDELIG® (idelalisib) Prescribing Information. Gilead Sciences, Inc., Foster City, CA, USA. Available at http://www.gilead.com/~/media/Files/pdfs/medicines/oncology/zydelig/zydelig_pi.pdf 2018;2016.

[23] Zelenetz AD, Barrientos JC, Brown JR, Coiffier B, Delgado J, Egyed M (2017). Idelalisib or placebo in combination with bendamustine and rituximab in patients with relapsed or refractory chronic lymphocytic leukaemia: interim results from a phase 3, randomised, double-blind, placebo-controlled trial. Lancet Oncol.

[24] Brucker Penny S., Yost Kathleen, Cashy John, Webster Kimberly, Cella David (2005). General Population and Cancer Patient Norms for the Functional Assessment of Cancer Therapy-General (FACT-G). Evaluation & the Health Professions.

[25] Cella D F, Tulsky D S, Gray G, Sarafian B, Linn E, Bonomi A, Silberman M, Yellen S B, Winicour P, Brannon J (1993). The Functional Assessment of Cancer Therapy scale: development and validation of the general measure. Journal of Clinical Oncology.

[26] Victorson David, Barocas Joshua, Song Juliette, Cella David (2008). Reliability across studies from the functional assessment of cancer therapy-general (FACT-G) and its subscales: a reliability generalization. Quality of Life Research.

[27] Webster K, Chivington K, Shonk C, Eremenco S, Yount S, Hahn E. Measuring quality of life (QOL) among patients with leukemia: the Functional Assessment of Cancer Therapy- Leukemia (FACT-LEU). Qual Life Res. 2002;11:Abstract 678.

[28] Cella David, Jensen Sally E., Webster Kimberly, Hongyan Du, Lai Jin-Shei, Rosen Steven, Tallman Martin S., Yount Susan (2012). Measuring Health-Related Quality of Life in Leukemia: The Functional Assessment of Cancer Therapy – Leukemia (FACT-Leu) Questionnaire. Value in Health.

[29] EuroQol G. EuroQol—a new facility for the measurement of health-related quality of life. Health Policy. 1990;16:199–208. 10.1016/0168-8510(90)90421-9 10109801

[30] Rabin R, de Charro F (2001). EQ-5D: a measure of health status from the EuroQol group. Ann Med.

[31] FACIT.org, 2015. http://www.facit.org/FACITOrg/Questionnaires. Accessed 09 Mar 2017

[32] EuroQol Research Foundation (2015). EQ-5D-5L User Guide. Basic information on how to use the EQ-5D-5L instrument version 2.1.

[33] Shaw JW, Johnson JA, Coons SJ (2005). US valuation of the EQ-5D health states: development and testing of the D1 valuation model. Med Care.

[34] Pickard AS, Neary MP, Cella D (2007). Estimation of minimally important differences in EQ-5D utility and VAS scores in cancer. Health Qual Life Outcomes.

[35] Furman RR, Sharman JP, Coutre SE, Cheson BD, Pagel JM, Hillmen P (2014). Idelalisib and rituximab in relapsed chronic lymphocytic leukemia. N Engl J Med.

[36] Jones JA, Robak T, Brown JR, Awan FT, Badoux X, Coutre S (2017). Efficacy and safety of idelalisib in combination with ofatumumab for previously treated chronic lymphocytic leukaemia: an open-label, randomised phase 3 trial. Lancet Haematol.

[37] Cramer Paula, Fraser Graeme, Santucci-Silva Rodrigo, Grosicki Sebastian, Dilhuydy Marie-Sarah, Janssens Ann, Loscertales Javier, Rule Simon, Goy Andre, Traina Shana, Chan Eric K. H., Diels Joris, Sengupta Nishan, Mahler Michelle, Salman Mariya, Howes Angela, Chanan-Khan Asher (2018). Improvement of fatigue, physical functioning, and well-being among patients with severe impairment at baseline receiving ibrutinib in combination with bendamustine and rituximab for relapsed chronic lymphocytic leukemia/small lymphocytic lymphoma in the HELIOS study. Leukemia & Lymphoma.

[38] Hillmen P, Janssens A, Babu KG, Kloczko J, Grosicki S, Manson S (2016). Health-related quality of life and patient-reported outcomes of ofatumumab plus chlorambucil versus chlorambucil monotherapy in the COMPLEMENT 1 trial of patients with previously untreated CLL. Acta Oncol.

