# Supplementary material for: Idelalisib addition has neutral to beneficial effects on quality of life in bendamustine/rituximab-treated patients: results of a phase 3, randomized, controlled trial
Source: Health Qual Life Outcomes. 2019 Nov 15;17:173. doi: 10.1186/s12955-019-1232-8 (PMC6858733; doi:10.1186/s12955-019-1232-8)
Supplement: Supplementary file 1 — Additional file 1: Table S1. Questionnaires used to assess health-related quality of life. Table S2. Compliance rates: FACT-Leu questionnaire. Table S3. Compliance rates: EQ-5D questionnaire. Table S4. Mixed-effects model analysis estimates (idelalisib/placebo) for functional assessment of cancer therapy using FACT-Leu. [file 12955_2019_1232_MOESM1_ESM.docx]

**Supplemental Materials**

**Table S1.** Questionnaires used to assess health-related quality of life

| **Instrument** | **Number of items** | **Score range^a^** | **Minimally important difference^b^** |
| --- | --- | --- | --- |
| **FACT-Leu questionnaire** |  |  |  |
| Leukemia-specific symptoms | 17 | 0–68 | 4–7 |
| Physical well-being | 7 | 0–28 | 2–3 |
| Social/family well-being | 7 | 0–28 | Not available |
| Functional well-being | 7 | 0–28 | 2–3 |
| Emotional well-being | 6 | 0–24 | 2 |
| **Composites** |  |  |  |
| Trial outcome index^c^ | 31 | 0–124 | 5–6 |
| FACT-Leu total^d^ | 44 | 0–176 | 6–12 |
| **EQ-5D utility index^e^** | 5 | –0.11 to 1 | 0.06–0.10 |
| **EQ-VAS** | 1 | 0–100 | 8–11 |

Analyzed in the ITT population.

^a^Higher scores reflect better HRQL and lower symptom burden.

^b^When examining differences for groups in a randomized trial, the lower end of the MID range was utilized.

^c^TOI = LeuS + PWB + FWB.

^d^FACT-Leu Total = LeuS + PWB + S/FWB + EWB + FWB.

^e^US index-based scores [1, 2].

EQ-5D, EuroQoL Five-Dimension; EQ-VAS, EuroQoL visual analog scale; FACT-Leu, Functional Assessment of Cancer Therapy – Leukemia; EWB, emotional well-being; FWB, functional well-being; HRQL, health-related quality of life; ITT, intent-to-treat; LeuS, leukemia-specific symptoms; MID, minimally important difference; PWB, physical well-being; S/FWB, social/family well-being; TOI, trial outcome index.

**Table S2.** Compliance rates: FACT-Leu questionnaire

| **Week** | | **N** | **PWB** | **S/FWB** | **EWB** | **FWB** | **LeuS** | **TOI** | **FACT-Leu Total Score** |
| --- | --- | --- | --- | --- | --- | --- | --- | --- | --- |
| **4** | Idelalisib | 203 | 193 (95.1%) | 193 (95.1%) | 191 (94.1%) | 192 (94.6%) | 193 (95.1%) | 192 (94.6%) | 192 (94.6%) |
|  | Placebo | 208 | 194 (93.3%) | 194 (93.3%) | 193 (92.8%) | 193 (92.8%) | 193 (92.8%) | 193 (92.8%) | 193 (92.8%) |
| **8** | Idelalisib | 197 | 180 (91.4%) | 180 (91.4%) | 179 (90.9%) | 179 (90.9%) | 179 (90.9%) | 179 (90.9%) | 179 (90.9%) |
|  | Placebo | 202 | 191 (94.6%) | 191 (94.6%) | 191 (94.6%) | 191 (94.6%) | 190 (94.1%) | 191 (94.6%) | 191 (94.6%) |
| **12** | Idelalisib | 194 | 178 (91.8%) | 178 (91.8%) | 178 (91.8%) | 178 (91.8%) | 177 (91.2%) | 178 (91.8%) | 178 (91.8%) |
|  | Placebo | 199 | 189 (95.0%) | 189 (95.0%) | 186 (93.5%) | 186 (93.5%) | 188 (94.5%) | 189 (95.0%) | 189 (95.0%) |
| **16** | Idelalisib | 189 | 167 (88.4%) | 166 (87.8%) | 167 (88.4%) | 167 (88.4%) | 167 (88.4%) | 167 (88.4%) | 167 (88.4%) |
|  | Placebo | 187 | 174 (93.0%) | 174 (93.0%) | 171 (91.4%) | 171 (91.4%) | 174 (93.0%) | 174 (93.0%) | 174 (93.0%) |
| **20** | Idelalisib | 182 | 154 (84.6%) | 154 (84.6%) | 154 (84.6%) | 154 (84.6%) | 153 (84.1%) | 154 (84.6%) | 154 (84.6%) |
|  | Placebo | 178 | 160 (89.9%) | 160 (89.9%) | 159 (89.3%) | 159 (89.3%) | 159 (89.3%) | 160 (89.9%) | 160 (89.9%) |
| **24** | Idelalisib | 173 | 156 (90.2%) | 155 (89.6%) | 156 (90.2%) | 156 (90.2%) | 155 (89.6%) | 156 (90.2%) | 156 (90.2%) |
|  | Placebo | 172 | 150 (87.2%) | 150 (87.2%) | 150 (87.2%) | 150 (87.2%) | 149 (86.6%) | 150 (87.2%) | 150 (87.2%) |
| **30** | Idelalisib | 164 | 147 (89.6%) | 147 (89.6%) | 146 (89.0%) | 146 (89.0%) | 147 (89.6%) | 147 (89.6%) | 147 (89.6%) |
|  | Placebo | 157 | 145 (92.4%) | 145 (92.4%) | 145 (92.4%) | 145 (92.4%) | 145 (92.4%) | 145 (92.4%) | 145 (92.4%) |
| **36** | Idelalisib | 154 | 139 (90.3%) | 139 (90.3%) | 138 (89.6%) | 138 (89.6%) | 137 (89.0%) | 139 (90.3%) | 139 (90.3%) |
|  | Placebo | 145 | 136 (93.8%) | 136 (93.8%) | 135 (93.1%) | 135 (93.1%) | 136 (93.8%) | 136 (93.8%) | 136 (93.8%) |
| **42** | Idelalisib | 147 | 134 (91.2%) | 134 (91.2%) | 133 (90.5%) | 133 (90.5%) | 134 (91.2%) | 134 (91.2%) | 134 (91.2%) |
|  | Placebo | 139 | 131 (94.2%) | 131 (94.2%) | 131 (94.2%) | 131 (94.2%) | 131 (94.2%) | 131 (94.2%) | 131 (94.2%) |
| **48** | Idelalisib | 140 | 129 (92.1%) | 129 (92.1%) | 128 (91.4%) | 128 (91.4%) | 128 (91.4%) | 129 (92.1%) | 129 (92.1%) |
|  | Placebo | 112 | 106 (94.6%) | 106 (94.6%) | 106 (94.6%) | 106 (94.6%) | 106 (94.6%) | 106 (94.6%) | 106 (94.6%) |
| **60** | Idelalisib | 134 | 125 (93.3%) | 125 (93.3%) | 123 (91.8%) | 123 (91.8%) | 124 (92.5%) | 125 (93.3%) | 125 (93.3%) |
|  | Placebo | 98 | 96 (98.0%) | 96 (98.0%) | 96 (98.0%) | 96 (98.0%) | 96 (98.0%) | 96 (98.0%) | 96 (98.0%) |
| **72** | Idelalisib | 90 | 78 (86.7%) | 77 (85.6%) | 78 (86.7%) | 78 (86.7%) | 78 (86.7%) | 78 (86.7%) | 78 (86.7%) |
|  | Placebo | 51 | 50 (98.0%) | 50 (98.0%) | 50 (98.0%) | 50 (98.0%) | 50 (98.0%) | 50 (98.0%) | 50 (98.0%) |
| **84** | Idelalisib | 56 | 47 (83.9%) | 47 (83.9%) | 47 (83.9%) | 47 (83.9%) | 47 (83.9%) | 47 (83.9%) | 47 (83.9%) |
|  | Placebo | 25 | 21 (84.0%) | 21 (84.0%) | 21 (84.0%) | 21 (84.0%) | 21 (84.0%) | 21 (84.0%) | 21 (84.0%) |
| **96** | Idelalisib | 38 | 34 (89.5%) | 34 (89.5%) | 34 (89.5%) | 34 (89.5%) | 34 (89.5%) | 34 (89.5%) | 34 (89.5%) |
|  | Placebo | 16 | 14 (87.5%) | 14 (87.5%) | 14 (87.5%) | 14 (87.5%) | 14 (87.5%) | 14 (87.5%) | 14 (87.5%) |
| **108** | Idelalisib | 26 | 23 (88.5%) | 23 (88.5%) | 23 (88.5%) | 23 (88.5%) | 23 (88.5%) | 23 (88.5%) | 23 (88.5%) |
|  | Placebo | 7 | 7 (100.0%) | 7 (100.0%) | 7 (100.0%) | 7 (100.0%) | 7 (100.0%) | 7 (100.0%) | 7 (100.0%) |
| **120** | Idelalisib | 11 | 9 (81.8%) | 9 (81.8%) | 9 (81.8%) | 9 (81.8%) | 9 (81.8%) | 9 (81.8%) | 9 (81.8%) |
|  | Placebo | 2 | 2 (100.0%) | 2 (100.0%) | 2 (100.0%) | 2 (100.0%) | 2 (100.0%) | 2 (100.0%) | 2 (100.0%) |
| **132** | Idelalisib | 4 | 2 (50.0%) | 2 (50.0%) | 2 (50.0%) | 2 (50.0%) | 2 (50.0%) | 2 (50.0%) | 2 (50.0%) |
|  | Placebo | 0 | 0 | 0 | 0 | 0 | 0 | 0 | 0 |
| **144** | Idelalisib | 2 | 1 (50.0%) | 1 (50.0%) | 1 (50.0%) | 1 (50.0%) | 1 (50.0%) | 1 (50.0%) | 1 (50.0%) |
|  | Placebo | 0 | 0 | 0 | 0 | 0 | 0 | 0 | 0 |

Presented as n (%).

FACT-Leu, Functional Assessment of Cancer Therapy – Leukemia; EWB, emotional well-being; FWB, functional well-being; LeuS, leukemia-specific symptoms; PWB, physical well-being; S/FWB, social/family well-being; TOI, trial outcome index.

**Table S3.** Compliance rates: EQ-5D questionnaire

| **Week** | | **N** | **Anxiety/ Depression** | **Mobility** | | **Pain/ Discomfort** | | **Self-Care** | | **Usual Activities** | | **VAS** | | **Overall Compliance** | |
| --- | --- | --- | --- | --- | --- | --- | --- | --- | --- | --- | --- | --- | --- | --- | --- |
| **4** | Idelalisib | 203 | 189 (93.1%) | 191 (94.1%) | | 188 (92.6%) | | 191 (94.1%) | | 191 (94.1%) | | 186 (91.6%) | | 191 (94.1%) | |
|  | Placebo | 208 | 195 (93.8%) | 195 (93.8%) | | 195 (93.8%) | | 193 (92.8%) | | 194 (93.3%) | | 191 (91.8%) | | 195 (93.8%) | |
| **8** | Idelalisib | 197 | 176 (89.3%) | 176 (89.3%) | | 176 (89.3%) | | 176 (89.3%) | | 176 (89.3%) | | 172 (87.3%) | | 177 (89.8%) | |
|  | Placebo | 202 | 188 (93.1%) | 188 (93.1%) | | 188 (93.1%) | | 188 (93.1%) | | 188 (93.1%) | | 186 (92.1%) | | 190 (94.1%) | |
| **12** | Idelalisib | 194 | 173 (89.2%) | 176 (90.7%) | | 176 (90.7%) | | 175 (90.2%) | | 176 (90.7%) | | 175 (90.2%) | | 176 (90.7%) | |
|  | Placebo | 199 | 189 (95.0%) | 189 (95.0%) | | 188 (94.5%) | | 188 (94.5%) | | 188 (94.5%) | | 187 (94.0%) | | 189 (95.0%) | |
| **16** | Idelalisib | 189 | 165 (87.3%) | 166 (87.8%) | | 166 (87.8%) | | 164 (86.8%) | | 164 (86.8%) | | 163 (86.2%) | | 166 (87.8%) | |
|  | Placebo | 187 | 172 (92.0%) | 172 (92.0%) | | 172 (92.0%) | | 171 (91.4%) | | 170 (90.9%) | | 171 (91.4%) | | 174 (93.0%) | |
| **20** | Idelalisib | 182 | 155 (85.2%) | 155 (85.2%) | | 155 (85.2%) | | 155 (85.2%) | | 155 (85.2%) | | 153 (84.1%) | | 155 (85.2%) | |
|  | Placebo | 178 | 159 (89.3%) | 160 (89.9%) | | 160 (89.9%) | | 160 (89.9%) | | 160 (89.9%) | | 160 (89.9%) | | 160 (89.9%) | |
| **24** | Idelalisib | 173 | 155 (89.6%) | 155 (89.6%) | | 155 (89.6%) | | 155 (89.6%) | | 154 (89.0%) | | 152 (87.9%) | | 155 (89.6%) | |
|  | Placebo | 172 | 151 (87.8%) | 150 (87.2%) | | 151 (87.8%) | | 151 (87.8%) | | 151 (87.8%) | | 148 (86.0%) | | 151 (87.8%) | |
| **30** | Idelalisib | 164 | 146 (89.0%) | 145 (88.4%) | | 144 (87.8%) | | 145 (88.4%) | | 144 (87.8%) | | 143 (87.2%) | | 146 (89.0%) | |
|  | Placebo | 157 | 145 (92.4%) | 145 (92.4%) | | 145 (92.4%) | | 145 (92.4%) | | 145 (92.4%) | | 145 (92.4%) | | 145 (92.4%) | |
| **36** | Idelalisib | 154 | 139 (90.3%) | 139 (90.3%) | | 139 (90.3%) | | 139 (90.3%) | | 138 (89.6%) | | 139 (90.3%) | | 140 (90.9%) | |
|  | Placebo | 145 | 134 (92.4%) | 134 (92.4%) | | 134 (92.4%) | | 134 (92.4%) | | 134 (92.4%) | | 135 (93.1%) | | 136 (93.8%) | |
| **42** | Idelalisib | 147 | 132 (89.8%) | 133 (90.5%) | | 134 (91.2%) | | 133 (90.5%) | | 133 (90.5%) | | 133 (90.5%) | | 134 (91.2%) | |
|  | Placebo | 139 | 130 (93.5%) | 131 (94.2%) | | 131 (94.2%) | | 131 (94.2%) | | 129 (92.8%) | | 130 (93.5%) | | 131 (94.2%) | |
| **48** | Idelalisib | 140 | 128 (91.4%) | 128 (91.4%) | | 128 (91.4%) | | 128 (91.4%) | | 128 (91.4%) | | 126 (90.0%) | | 128 (91.4%) | |
|  | Placebo | 112 | 105 (93.8%) | 105 (93.8%) | | 104 (92.9%) | | 105 (93.8%) | | 104 (92.9%) | | 106 (94.6%) | | 106 (94.6%) | |
| **60** | Idelalisib | 134 | 125 (93.3%) | 125 (93.3%) | | 125 (93.3%) | | 125 (93.3%) | | 125 (93.3%) | | 123 (91.8%) | | 125 (93.3%) | |
|  | Placebo | 98 | 96 (98.0%) | 96 (98.0%) | | 96 (98.0%) | | 96 (98.0%) | | 96 (98.0%) | | 96 (98.0%) | | 96 (98.0%) | |
| **72** | Idelalisib | 90 | 77 (85.6%) | 77 (85.6%) | | 77 (85.6%) | | 77 (85.6%) | | 77 (85.6%) | | 78 (86.7%) | | 78 (86.7%) | |
|  | Placebo | 51 | 50 (98.0%) | 50 (98.0%) | | 50 (98.0%) | | 50 (98.0%) | | 50 (98.0%) | | 50 (98.0%) | | 50 (98.0%) | |
| **84** | Idelalisib | 56 | 48 (85.7%) | 48 (85.7%) | | 48 (85.7%) | | 48 (85.7%) | | 48 (85.7%) | | 47 (83.9%) | | 48 (85.7%) | |
|  | Placebo | 25 | 21 (84.0%) | 21 (84.0%) | | 21 (84.0%) | | 21 (84.0%) | | 21 (84.0%) | | 21 (84.0%) | | 21 (84.0%) | |
| **96** | Idelalisib | 38 | 33 (86.8%) | 33 (86.8%) | | 33 (86.8%) | | 33 (86.8%) | | 33 (86.8%) | | 34 (89.5%) | | 34 (89.5%) | |
|  | Placebo | 16 | 14 (87.5%) | 14 (87.5%) | | 14 (87.5%) | | 14 (87.5%) | | 14 (87.5%) | | 14 (87.5%) | | 14 (87.5%) | |
| **108** | Idelalisib | 26 | 22 (84.6%) | 22 (84.6%) | | 22 (84.6%) | | 22 (84.6%) | | 22 (84.6%) | | 22 (84.6%) | | 22 (84.6%) | |
|  | Placebo | 7 | 7 (100.0%) | 7 (100.0%) | | 7 (100.0%) | | 7 (100.0%) | | 7 (100.0%) | | 7 (100.0%) | | 7 (100.0%) | |
| **120** | Idelalisib | 11 | 9 (81.8%) | 9 (81.8%) | | 9 (81.8%) | | 9 (81.8%) | | 9 (81.8%) | | 9 (81.8%) | | 9 (81.8%) | |
|  | Placebo | 2 | 2 (100.0%) | 2 (100.0%) | | 2 (100.0%) | | 2 (100.0%) | | 2 (100.0%) | | 2 (100.0%) | | 2 (100.0%) | |
| **132** | Idelalisib | 4 | 2 (50.0%) | | 2 (50.0%) | | 2 (50.0%) | | 2 (50.0%) | | 2 (50.0%) | | 2 (50.0%) | | 2 (50.0%) |
|  | Placebo | 0 | 0 | | 0 | | 0 | | 0 | | 0 | | 0 | | 0 |
| **144** | Idelalisib | 2 | 1 (50.0%) | | 1 (50.0%) | | 1 (50.0%) | | 1 (50.0%) | | 1 (50.0%) | | 1 (50.0%) | | 1 (50.0%) |
|  | Placebo | 0 | 0 | | 0 | | 0 | | 0 | | 0 | | 0 | | 0 |

Presented as n (%).

EQ-5D, EuroQoL Five-Dimension; VAS, visual analog scale.

**Table S4.** Mixed-effects model analysis estimates (idelalisib/placebo) for functional assessment of cancer therapy using FACT-Leu

| **Treatment difference^a^**  **LSM (95% CI)** | | | | | | | |
| --- | --- | --- | --- | --- | --- | --- | --- |
|  | **LeuS** | **PWB** | **FWB** | **EWB** | **S/FWB** | **TOI** | **FACT-Leu Total** |
| **Week 4**  ***P*-value** | 1.42 (−0.26, 3.09)  0.0968 | 0.49 (−0.39, 1.38)  0.2747 | 0.16 (−0.77, 1.10)  0.7326 | 0.37 (−0.35, 1.08)  0.3165 | 0.84 (−0.01, 1.68)  **0.0525** | 2.11 (−0.69, 4.91)  0.1399 | 3.70 (0.28, 7.13)  **0.0343** |
| **Week 8**  ***P*-value** | 0.77 (−1.17, 2.71)  0.4342 | 0.11 (−0.88, 1.10)  0.8244 | −0.19 (−1.22, 0.83)  0.7116 | −0.15 (−0.94, 0.64)  0.7179 | 0.51 (−0.35, 1.36)  0.2441 | 0.84 (−2.56, 4.24)  0.6273 | 1.46 (−2.70, 5.62)  0.4896 |
| **Week 12**  ***P*-value** | 0.51 (−1.57, 2.60)  0.6278 | −0.05 (−1.08, 0.98)  0.9211 | −0.44 (−1.60, 0.72)  0.4564 | −0.29 (−1.20, 0.62)  0.5300 | 0.28 (−0.58, 1.15)  0.5219 | −0.21 (−3.91, 3.49)  0.9111 | 0.01 (−4.59, 4.61)  0.9958 |
| **Week 16**  ***P*-value** | 0.45 (−1.52, 2.43)  0.6518 | 0.15 (−0.87, 1.17)  0.7708 | −0.46 (−1.57, 0.66)  0.4208 | −0.37 (−1.18, 0.43)  0.3641 | 0.58 (−0.33, 1.48)  0.2111 | 0.04 (−3.46, 3.53)  0.9831 | 0.22 (−4.06, 4.51)  0.9183 |
| **Week 20**  ***P*-value** | 1.56 (−0.43, 3.55)  0.1241 | 0.47 (−0.61, 1.56)  0.3938 | 0.07 (−1.06, 1.20)  0.9053 | –0.20 (−1.07, 0.66)  0.6419 | 0.41 (−0.49, 1.31)  0.3724 | 2.10 (−1.55, 5.76)  0.2588 | 2.46 (−2.00, 6.93)  0.2790 |
| **Week 24**  ***P*-value** | 0.93 (−1.19, 3.04)  0.3901 | 0.04 (−1.14, 1.21)  0.9521 | −0.94 (−2.10, 0.23)  0.1150 | −0.31 (−1.21, 0.59)  0.4990 | 0.24 (−0.65, 1.13)  0.5953 | 0.16 (−3.75, 4.06)  0.9370 | 0.40 (−4.36, 5.17)  0.8678 |
| **Week 30**  ***P*-value** | 0.52 (−1.63, 2.68)  0.6348 | −0.04 (−1.14, 1.06)  0.9378 | −0.20 (−1.45, 1.06)  0.7603 | −0.13 (−1.05, 0.79)  0.7760 | 0.26 (−0.69, 1.20)  0.5918 | 0.41 (−3.52, 4.33)  0.8392 | 0.76 (−4.07, 5.59)  0.7572 |
| **Week 36**  ***P*-value** | 0.93 (−1.18, 3.04)  0.3863 | 0.11 (−1.04, 1.26)  0.8480 | −0.05 (−1.20, 1.10)  0.9317 | −0.34 (−1.27, 0.60)  0.4781 | 0.80 (−0.10, 1.70)  0.0810 | 1.20 (−2.56, 4.96)  0.5313 | 1.68 (−2.98, 6.33)  0.4789 |
| **Week 42**  ***P*-value** | 0.42 (−1.72, 2.57)  0.6966 | −0.50 (−1.67, 0.67)  0.3986 | −0.21 (−1.38, 0.95)  0.7191 | −0.30 (−1.26, 0.66)  0.5354 | 0.30 (−0.70, 1.30)  0.5584 | −0.12 (−4.05, 3.81)  0.9527 | −0.21 (−5.03, 4.61)  0.9313 |
| **Week 48**  ***P*-value** | 1.48 (−0.84, 3.79)  0.2112 | 0.13 (−0.96, 1.23)  0.8104 | −0.10 (−1.34, 1.15)  0.8772 | −0.10 (−0.99, 0.79)  0.8221 | 0.27 (−0.66, 1.21)  0.5668 | 1.65 (−2.40, 5.70)  0.4241 | 1.73 (−3.35, 6.82)  0.5030 |
| **Week 60**  ***P*-value** | 2.78 (0.45, 5.10)  **0.0192** | 0.54 (−0.69, 1.77)  0.3895 | −0.38 (−1.70, 0.94)  0.5738 | −0.13 (−0.98, 0.72)  0.7667 | 0.12 (−0.91, 1.15)  0.8181 | 2.88 (−1.48, 7.24)  0.1944 | 3.11 (−2.20, 8.41)  0.2505 |
| **Week 72**  ***P*-value** | 1.20 (−1.42, 3.82)  0.3676 | −0.59 (−2.01, 0.82)  0.4105 | 0.14 (−1.32, 1.61)  0.8470 | −0.13 (−1.33, 1.07)  0.8268 | 0.51 (−0.64, 1.67)  0.3839 | 1.23 (−3.64, 6.10)  0.6197 | 2.01 (−3.87, 7.90)  0.5020 |
| **Week 84**  ***P*-value** | 2.48 (−0.82, 5.78)  0.1399 | −0.73 (−2.24, 0.79)  0.3465 | −0.32 (−2.05, 1.41)  0.7140 | −0.01 (−1.52, 1.50)  0.9912 | 0.57 (−0.62, 1.76)  0.3448 | 2.42 (−3.23, 8.07)  0.4005 | 2.40 (−4.48, 9.28)  0.4937 |

Analyzed in the ITT population. Bolded numbers are statistically significant (*P* ≤0.5).

^a^Treatment difference was calculated by subtracting the LSM change from baseline in the placebo group from the LSM change from baseline in the idelalisib group.

CI, Confidence interval; EWB, emotional well-being; FACT-Leu, Functional Assessment of Cancer Therapy – Leukemia; FWB, functional well-being; ITT, intent-to-treat; LeuS, leukemia-specific symptoms; LSM, least squares mean; PWB, physical well-being; S/FWB, social/family well-being; TOI, trial outcome index.

**Supplemental references**

1. Pickard AS, Neary MP, Cella D. Estimation of minimally important differences in EQ-5D utility and VAS scores in cancer. Health Qual Life Outcomes. 2007;5:70.

2. Shaw JW, Johnson JA, Coons SJ. US valuation of the EQ-5D health states: development and testing of the D1 valuation model. Med Care. 2005;43:203-20.
